# Characterization of Health Beneficial Components in Discarded Leaves of Three Escarole (*Cichorium endivia* L.) Cultivar and Study of Their Antioxidant and Anti-Inflammatory Activities

**DOI:** 10.3390/antiox12071402

**Published:** 2023-07-08

**Authors:** Giuliana Donadio, Maria Laura Bellone, Francesca Mensitieri, Valentina Parisi, Valentina Santoro, Maria Vitiello, Fabrizio Dal Piaz, Nunziatina De Tommasi

**Affiliations:** 1 Dipartimento di Farmacia, Università degli Studi di Salerno, Via Giovanni Paolo II 132, 84084 Fisciano, SA, Italy; gdonadio@unisa.it (G.D.); mbellone@unisa.it (M.L.B.); vparisi@unisa.it (V.P.); vsantoro@unisa.it (V.S.); 2 Bioactiplant SRL, Via Dell’Ateneo Lucano 10, 85100 Potenza, PZ, Italy; 3 Dipartimento di Medicina, Chirurgia e Odontoiatria “Scuola Medica Salernitana”, Università degli Studi di Salerno, Via Salvador Allende 43, 84081 Baronissi, SA, Italy; fmensitieri@unisa.it (F.M.); fdalpiaz@unisa.it (F.D.P.); 4 National Biodiversity Future Center (NBFC), 90133 Palermo, PA, Italy; 5 Dipartimento di Farmacia, Università di Pisa, Via Bonanno Pisano 12, 56126 Pisa, PI, Italy; maria.vitiello@phd.unipi.it

**Keywords:** *Cichorium endivia* L., cultivar, NMR, HRESIMS, anti-inflammatory, antioxidant, green extraction, waste valorisation

## Abstract

Plants of genus *Cichorium* (Asteraceae) can be used as vegetables with higher nutritional value and as medicinal plants. This genus has beneficial properties owing to the presence of a number of specialized metabolites such as alkaloids, sesquiterpene lactones, coumarins, unsaturated fatty acids, flavonoids, saponins, and tannins. *Cichorium endivia* L., known as escarole, has achieved a common food status due to its nutritionary value, bitter taste, and the presence of healthy components, and is eaten cooked or raw in salads. Presently, wastes derived from the horticultural crops supply chain are generated in very large amounts. Vegetable waste comprises the discarded leaves of food sources produced during collection, handling, transportation, and processing. The external leaves of *Cichorium endivia* L. are a horticultural crop that is discarded. In this work, the phytochemical profile, antioxidant, and anti-inflammatory activities of hydroalcoholic extract obtained from discarded leaves of three cultivars of escarole (*C. endivia* var. *crispum* ‘Capriccio’, *C. endivia* var. *latifolium* ‘Performance’ and ‘Leonida’) typical horticultural crop of the Campania region were investigated. In order to describe a metabolite profile of *C. endivia* cultivars, the extracts were analysed by HR/ESI/Qexactive/MS/MS and NMR. The careful analysis of the accurate masses, the ESI/MS spectra, and the ^1^H NMR chemical shifts allowed for the identification of small molecules belonging to phenolic, flavonoid, sesquiterpene, amino acids, and unsaturated fatty acid classes. In addition, the antioxidant potential of the extracts was evaluated using cell-free and cell-based assays, as well as their cytotoxic and anti-inflammatory activity. All the extracts showed similar radical-scavenging ability while significant differences between the three investigated cultivars emerged in the cell-based assays. The obtained data were ascribed to the content of polyphenols and sesquiterpenes in the extracts. Accordingly, *C. endivia* by-products can be deemed an interesting material for healthy product formulations.

## 1. Introduction

Cichorieae tribe (Asteraceae) is comprised of perennial herbs, including many cultivars that differ in color, size, and shape. These herbs are widely used as edible food and are commercially relevant [1]. Cichoriae are rich in nutritional components, such as minerals, vitamins, fatty acids, amino acids, carbohydrates, and dietary fibers found in roots and leaves [2]. In addition, many specialized metabolites were detected in plants belonging to this tribe, including phenolic acids (caffeoylquinic acids, chicoric acid, caftaric acid, ferulic acid), flavonoids (quercetin and luteolin derivatives, catechin, and in the red varieties “Radicchio rosso” and “Lattuga rossa” anthocyanins) [3], carotenoids, and sesquiterpene lactones, with the latter located mainly in the root [4,5,6]. All these compounds are responsible for the healthy properties (antimicrobial, antioxidant, anti-inflammatory, hepatoprotective, and antidiabetic) of chicory on human health [7,8,9,10]. *Cichorium endivia* L., known as escalora, is a biennial or perennial herb and is one of the most common leafy vegetables [11]. *Cichorium endivia* var. *crispum* and var. *latifolium* are eaten worldwide as fresh or marginally processed salads, providing healthy components. Escarole *crispum* is the curly endive that bears green leaves with a narrow central vein and a septate blade with engraved margins, while *latifolium*, the smooth-leafed types, produce lighter green leaves with a large midrib, a broad and slightly lobed lamina, and dentate margins [12].

This species presents an annual life cycle, and it blooms prolongs from May to August in the Mediterranean areas [12] and is of great value due to its medicinal properties. Its beneficial effect is due to the presence of several bioactive compounds such as alkaloids, sesquiterpene lactones, coumarins, vitamins, unsaturated fatty acids, flavonoids, saponins, and tannins [5,6,7,8]. As reported in the literature, antioxidant activities were found in the polyphenolic fraction of *Cichorium* extracts due the presence of chlorogenic acid derivatives. Additionally, *C. endivia* sesquiterpenes displayed numerous bioactivities; in vivo data showed analgesic and sedative effects for lactucin and lactucopicrin [13]. Additionally, an inhibitory effect to DNA binding of nuclear factor (NF)kB and cyclooxygenase (COX)-2 protein expression was established for 8-deoxylactucin [14].

*C. endivia* is included in the so-called minimally processed products (MPP), as it is a fresh vegetable ready for consumption. However, its use generates a high amount of waste product. The waste and by-products of the horticultural crops chain are arousing more and more interest; in fact, according to the United Nations [15], the world population is expected to increase from 7.7 billion (2019) to 9.7 billion in 2050, and the annual production of waste, following the current trend, will increase by 70% in the next 40 years [16]. Studies have shown that waste of vegetable processing consists of large amounts of proteins, sugars, and lipids, together with specialized metabolites and, therefore, could be an abundant and cheap source of high value-added products that are potentially useful as health and food products. The pharmaceutical, cosmetic, and food industries could benefit from the use of natural bioactive compounds recovered from waste to produce nutraceuticals and functional products.

Some *C. indivia* varieties, cultivars, and landraces are registered in the “National List of Traditional Agri-Food Products” of the Ministry of Agricultural, Food, and Forestry Policies. The production of total extracts or constituents from regional crops to be employed in agro- or in pharmaceutical industry currently represents a chance to obtain a sustainable territorial development in the vision of the European Green Deal.

This research was focused on evaluating the effective suitability of *C. endivia* industrial waste as a source of bioactive compounds for animal and human use for food and/or health purposes. Therefore, discarded leaves of three cultivars of escarole cultivated in Campania region (*C. endivia* var. *crispum* ‘Capriccio’, *C. endivia* var. *latifolium* ‘Performance’, and ‘Leonida’) were selected and their phytochemical composition was studied by way of a liquid chromatography-mass spectrometry (LC-MS)-based and NMR targeted approach. Furthermore, considering the interesting biological activity reported for this genus, the three cultivars’ extracts were studied both for radical scavenger activity by DPPH and ABTS+-based assays and for anti-inflammatory potential by evaluating their effect on the secretion of IL-6, a typical marker of inflammation in human macrophages.

## 2. Materials and Methods

### 2.1. Plant Materials

The discarded leaves of three cultivars of *Cichorium endivia* L. were studied: var. *crispum* ‘Capriccio’, var. *latifolium* ‘Leonida’, and ‘Performance’. The three cultivars were obtained from Finagricola—Soc. Coop. Viale Spagna, 8—Z.I. 84091 Battipaglia (SA)—Italy. These leaves were divided into smaller pieces and then frozen at −20 °C to minimize the degradation processes until analysis.

### 2.2. Extraction Procedure

Previously frozen leaves (100 g) of the three escarole cultivars were extracted by ultrasound technique using a Q500 immersion Sonicator (QSonica, Newtown, CT, USA). 600 mL of EtOH/H_2_O in an 80:20 (*v*/*v*) ratio were added to the plant material. The extracts thus obtained were filtered and concentrated under vacuum with a rotavapor at a temperature of 30 °C and were freeze-dried. The flasks were subsequently weighed in order to evaluate their extraction yield (3–4%). The lyophilized extracts were transferred into vials and stored at room temperature.

### 2.3. NMR Analysis

#### 2.3.1. Sample Preparation

Three replicates for each cultivar were used, with a total of 6 samples. Briefly, 5 mg of dried extracts were dissolved in 0.9 mL of a solution composed by 650 µL CD_3_OD (99.95%) and 250 µL KH_2_PO_4_ buffer in D_2_O (pH 6.0), containing 0.01% 3-(trimethylsilyl)propionic-2,2,3,3-d4 acid sodium salt (TSP). After centrifugation at 13,000 rpm for 10 min, the clear supernatants (600 μL) were transferred into NMR tubes for further analysis.

#### 2.3.2. NMR Spectroscopy and Processing

The NMR spectra were acquired on Bruker Avance 600 spectrometer (Bruker BioSpin GmBH, Rheinstetten, Germany) equipped with a 5 mm probe operating at 298 K. ^1^H NMR experiments were recorded using a CPMG (cpmgpr1d) pulse sequence with water signal suppression. The used acquisition parameters were as follows: 32 K data points, 12,019 Hz (20 ppm) spectral width, 4 dummy and 64 scans, a recycle delay of 4 s, and a fixed value for receiver gain for all samples. All spectra were acquired in triplicate. The metabolites were annotated based on the comparison of their ^1^H NMR spectra to those of the reference compounds in the CL libraries or in the literature data.

### 2.4. Mass Spectrometry Analyses

#### 2.4.1. Qualitative Analyses

The hydroalcoholic extracts of three *C. endivia*, ‘Performance’, ‘Leonida’, and ‘Capriccio’, were subjected to LC-MS/MS analysis using an apparatus composed by a Thermo Scientific UltiMate 3000 UHPLC system (Thermo Fischer Scientific Inc., Darmstadt, Germany) coupled with a high-resolution mass spectrometry Q Exactive™ Hybrid Quadrupole-Orbitrap™ (Thermo Fischer Scientific Inc., Darmstadt, Germany). Compound separation was achieved using a Luna^®^ C_18_ 150 × 2 mm, 3 µm (100 Å) column (Phenomenex^®^, Castel Maggiore, Bologna, Italy), a mobile phase consisting of 0.1% (*v*/*v*) formic acid in water (eluent A) and acetonitrile (eluent B), and a linear gradient from 5 to 50% of eluent B over 30 min with a flow rate of 0.2 mL/min.

Mass Spectrometer operated in both positive and negative ion mode. The identification of specialized metabolites was based on their accurate measured mass, acquired MS/MS spectra, comparison with the literature data, and, if available, injection of pure standards.

#### 2.4.2. Quantitative Analyses

The quantitative determination of the most abundant sesquiterpenes was achieved with a Thermo Scientific UltiMate 3000 UHPLC system coupled with a high-resolution mass spectrometry Q Exactive™ Hybrid Quadrupole-Orbitrap™ (Thermo Fischer Scientific Inc., Darmstadt, Germany). A C_18_ column, Luna^®^ 150 × 2 mm, 3 µm (100 Å) (Phenomenex^®^, Castel Maggiore, Bologna, Italy) with a mobile phase consisting of 0.1% (*v*/*v*) formic acid in water (eluent A) and acetonitrile (eluent B) and a linear gradient from 5% of B and reached 50% in 25 min. were used. The main flavonoid compounds were quantified with an ABSCIEX API 6500 QTRAP^®^ Mass Spectrometer coupled with a Nexera X2 UPLC Shimadzu system operating in positive and negative ion modes. Compound separation was achieved using a Luna Omega^®^ C_18_ 100 × 2 mm, 1.7 µm (100 Å) column (Phenomenex^®^, Castel Maggiore, Bologna, Italy), a mobile phase consisting of 0.1% (*v*/*v*) formic acid in water (eluent A) and acetonitrile (eluent B) and a gradient from 5% of B and reached 50% in 15 min, % of B. The mass spectrometer operated in MRM mode, using parameters optimized for each analyte. Sesquiterpene compounds were quantified as lactucin equivalent, while kaempferol derivatives, the most abundant flavonoids, were quantified with the respective aglycon. The linearity of the instrumental response was verified for each compound.

### 2.5. Antioxidant Assay

#### 2.5.1. The DPPH (2,2′-Diphenyl-1-picrylhydrazyl Radical) Assay

The DPPH (2,2′-diphenyl-1-picrylhydrazyl radical) assay is based on spectrophotometric monitoring of the reaction between radical cation and hydrogen donors. Free radical scavenging ability of the extracts was tested by DPPH (2,2′-diphenyl-1-picrylhydrazyl radical) radical scavenging assay [17]. DPPH produces a violet/purple colour in methanol and changes the colour to yellow in the presence of antioxidants. A stock solution of 10 mM DPPH was prepared in methanol and diluted until an absorbance of 1 OD at 515 nm was reached. Samples were tested in the presence of a 0.15 mM final concentration of DPPH in 100% methanol. Extracts were diluted between 500 and 2000-fold; the reaction was allowed to proceed for 30 min in the dark at room temperature, and then the decrease in absorbance at 515 nm was measured. Several Trolox µM concentrations (0–100 µM-X-axis) were incubated with DPPH, and their absorbance was measured at 515 nm (*Y*-axis). A calibration curve was constructed with Trolox concentrations and Abs at 515 nm. All determinations were carried out in triplicate. Millimolar concentrations of Trolox equivalents (TE) of dry extract were quantified using the linear regression equation as follows: extract TE µM = [(Abs 515 nm−1.1170)/(−0.004)]. Following that, the appropriate dilution factor was applied to calculate the millimolar TE of the extract at 10 mg/mL.

#### 2.5.2. ABTS Assay Radical Cation Decolorization Assay

ABTS assay radical cation decolorization assay is based on the reduction of ABTS+• radicals by antioxidants [18]. For the study, the ABTS+• solution was diluted in PBS (Phosphate Buffer Saline) to an absorbance of 0.7 (±0.02) at 734 nm. After the addition of 100 μL of extract solutions to 100 μL of ABTS+• solution, the absorbance reading was taken at 30 °C for 10 min after initial mixing. All solutions were used on the day of preparation, and all determinations were carried out in triplicate. Samples were compared to known concentrations of Trolox standards, a water-soluble analog of tocopherol (Vitamin E), which is a very strong antioxidant that is commonly used to measure antioxidant capacity. Different Trolox µM concentrations (0–50 µM-*X*-axis) were incubated in the presence of the ABTS radical, and its absorbance was measured at 734 nm (*Y*-axis). Micromolar concentrations of Trolox equivalents (TE) of dry extract were quantified using the linear regression equation as follows: extract TE µM = [(Abs 734 nm−0.7314)/(−0.0145)]. Following that, the appropriate dilution factor was applied to calculate the millimolar TE of the extract at 10 mg/mL.

#### 2.5.3. H_2_O_2_ Scavenging Activity Using HRP Assay

H_2_O_2_ scavenging activity of natural extracts was assessed using the horseradish peroxidase (HRP) assay. Pyrogallol conversion in purpurgallin chromogenic product in the presence of H_2_O_2_ was followed spectrophotometrically at 420 nm, according to the reaction:

$$Pyrogallol+H_{2}O_{2}\overset{HRP}{\to }Purpurgallin+H_{2}O$$

The optimal reaction conditions were set up following manufacturer instructions (Enzymatic Assay of Peroxidase (EC 1.11.1.7)—Sigma Aldrich. St. Louis, MO, USA). Assays were carried out in 1 mL containing 14 mM potassium phosphate buffer at pH 6. 0.75 U of HRP peroxidase (Peroxidase from horseradish, type VI, Sigma Aldrich), which were used in presence of 5 mg/mL of pyrogallol and 0.175 mM H_2_O_2_. Reactions were incubated for 1.30 h at RT and then H_2_O_2_ concentration was quantified at the assay end-point by assessing purpurgallin concentration through its molar extinction coefficient (ε_420_ = 2640 M^−1^ cm^−1^). To assess *C. endivia* leaves and the extract-scavenging ability of H_2_O_2_, the extracts were pre-incubated in 14 mM potassium phosphate buffer at pH 6 in the presence of 0.375 mM H_2_O_2_ for 30 min. Concentrations of 500, 250, 125, 50, and 25 μg/mL were tested for each leaf extract. For each concentration of plant extract, time zero absorbance value at 420 nm was measured to correct the eventual colorimetric interference of leaves extracts. The assay controls were carried out by incubating H_2_O_2_ in potassium phosphate buffer, and H_2_O_2_ in potassium phosphate buffer in the presence of 30 U of catalase, as positive and negative controls, respectively. Pyrogallol and HRP were added and HRP assay was performed as described above. Purpurgallin absorbance subtracted from the time zero measurement was used for H_2_O_2_ quantification.

### 2.6. Cell Culture and Differentiation

THP-1 (human acute monocytic leukaemia cells) cells were cultured in RPMI 1640 and supplemented with 10% Fetal Bovine Serum (FBS), 100 mg/L streptomycin, and penicillin 100 IU/mL, and were maintained at 37 °C and 5% CO_2_. To obtain THP-1-derived macrophages M0, THP-1 were treated for 24 h with 100 nM of phorbol-12-myristate-13-acetate (PMA). Cell differentiation was enhanced by replacing the PMA-containing media with fresh media for 24 h.

### 2.7. Cell Viability Assay

THP-1 were seeded in 96-well plates (5 × 10^4^ cells/well) and differentiated into M0, as described above. The cells were treated with different concentrations of *C. endivia* cultivars extracts in the range 31.2–250 µg/mL for 24 h and 48 h. The number of viable cells was quantified by MTT [3-(4,5-dimethylthiazol-2-yl)-2,5-diphenyl tetrazolium bromide] assay. Absorption was measured at 550 nm using Multiskan GO (Thermo Scientific, Waltham, MA, USA). Experiments were performed in technical replicates.

### 2.8. Cellular Antioxidant Activity (CAA) Evaluation on THP-1 Derived Macrophages M0

OxiSelect Cellular Antioxidant Activity Assay Kit (Cell Biolabs Inc. Cat. No. STA-349, San Diego, CA, USA) was used to investigate the cellular antioxidant activity of escarole extracts. Firstly, THP-1 were seeded in 96-well plates (5 × 10^4^ cells/well) and differentiated into M0 as described above. The cells were treated with DCFH-DA probe solution and extract sample (50–100 µg/mL) and incubated for 1 h. Following that, after washing with DPBS 1X buffer, the free radical initiator solution was incubated and the plates were underwent fluorescence microplate reader analysis using 480 nm and 530 nm as excitation and emission wavelengths, respectively. The measurement was performed on the time interval 0–60 min with readings every 5 min. Obtained data were used to measure the AUC value (Area Under the Curve) for each sample and then the CAA value (Cellular Antioxidant Activity) was calculated as follows: [CAA Units = 100 − (AUCAntioxidant/AUCControl) × 100] [19]. Quercetin standard was used as a positive control. Experiments were performed in technical duplicates and in biological duplicates.

### 2.9. Cytokine Production and Enzyme-Linked Immunosorbent Assay (ELISA) on THP-1 Derived Macrophages M0

THP-1 were seeded in 6-well plates (1 × 10^6^ cells/well) and differentiated into M0 as described above. Following that, M0 cells were incubated with and without LPS (0.1 µg/mL) for 3 h and then treated with ‘Capriccio’, ‘Performance’ (100 µg/mL), and ‘Leonida’ (50 and 100 µg/mL) for 24 h. The conditioned medium was collected and analysed by Enzyme-Linked Immunosorbent Assay (ELISA). The assays were performed according to manufacturer instructions to quantify the release of inflammatory cytokine (IL-6). The values were normalized to the LPS sample and reported as percentages.

### 2.10. Superoxide Dismutase (SOD) Colorimetric Activity Kit

The effects of escarole extracts on SOD activity were evaluated using the K028-H1 kit (DetectX, Ann Arbor, MI, USA) and M0 cells. THP-1 cells were seeded in 12-well plates (4 × 10^5^ cells/well) and differentiated as described above. Following that, M0 cells were incubated with the three escarole extracts at a concentration of 100 µg/mL for 2 h or with the vehicle (control) and lysated. The SOD activity of each resulting protein mixture was then measured according to the manufacturer’s specifications and then compared to that of the control. The experiment was performed in technical duplicate. The amount of SOD1 in the cell lysates was measured by western blot analysis.

### 2.11. Western Blot Analysis

THP-1 were seeded in 6-well plates (1 × 10^6^ cells/well) and differentiated into M0 as described above. Following that, M0 cells were pre-treated with ‘Capriccio’, ‘Performance’, and ‘Leonida’ (50 and 100 µg/mL) for 1 h. The medium was changed and Free Radical Initiator in PBS1X were added for 1 h. After incubation, the cells were lysed with RIPA buffer (20 mM Tris-HCl pH 7.5, 150 mM NaCl, 1 mM EDTA, 1% NP-40, 1% sodium deoxycholate, 2.5 mM sodium pyrophosphate). Protein concentration was determined by a Bradford solution (Applichem, Darmstadt, Germany) using BSA as a standard. 30 µg of proteins were loaded on 12%-SDS-PAGE, transferred into nitrocellulose membranes, and immunoblotted with the SOD1 primary antibody (Enzo Life Sciences, Farmingdale, NY, USA) and with horseradish peroxidase-conjugated secondary antibody. Signals were visualized by enhanced chemiluminescence (Amersham Biosciences-GE Healthcare, New York, NY, USA). Densitometric analysis was carried out using the ImageJ software Java8.

### 2.12. Catalase Activity Assay

Catalase activity was evaluated by determining the H_2_O_2_ degradation catalysed by the enzyme in vitro according to the reaction:

H_2_O_2_ + 2 H_2_O    3 H_2_O + O_2_

After incubation with catalase, H_2_O_2_ consumption was assessed using a coupled HRP assay with pyrogallol, following the same protocol previously described. Briefly, 30 U of catalase (catalase from bovine liver, lyophilized, Sigma-Aldrich) were incubated in 0.9 mL of 14 mM potassium phosphate buffer pH 6 containing 0.75 mM H_2_O_2_, and in the absence (positive control) or presence of 500 μg/mL of leaf extracts for 1 h at RT. Negative control was performed by incubating 0.75 mM H_2_O_2_ without catalase. 5 mg/mL of pyrogallol and 0.75 U of HRP peroxidase were added to each reaction mixture and the latter were further incubated for 1.30 h at RT. Finally, H_2_O_2_ concentration was quantified through the HRP end-point assay previously described by assessing purpurgallin concentration through its molar extinction coefficient (ε_420_ = 2640 M^−1^ cm^−1^). For each plant extract reaction mixture, time zero absorbance value at 420 nm was measured to correct the eventual colorimetric interference of leaves extracts. Purpurgallin absorbance at 420 nm subtracted of the time 0 measurement was used for H_2_O_2_ quantification. The eventual inhibition of catalase activity was evaluated from the increased H_2_O_2_ concentration as compared to the positive control reaction (30 U catalase without extracts).

## 3. Results

### 3.1. Optimized Ultrasound-Assisted Extraction Procedure

**Figure 1:**
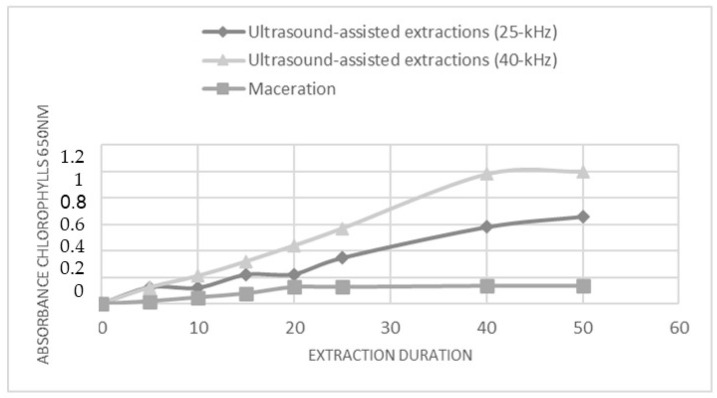
The effect of power ultrasound on *C. endivia* cultivars leaves. Comparison of chlorophyll extraction kinetics for UAE, US probe 25 kHz and 40 kHz, and for maceration.

The metabolites extraction from *C. endivia* cultivar leaf was carried out through maceration or ultrasound-assisted extractions (UAE). Extraction conditions such as type of solvent, temperature, duration, and frequency were optimized. The extraction processes were conducted using an ethanol (Et): water (Wt) mixture (80:20 *v*/*v*), and extraction times were up to 50 min. The solvent system was selected for its ability to extract and solubilize a wide range of primary and secondary metabolites and for its biocompatibility and low environmental impact. A short extraction time was chosen in order to implement an extraction procedure that was compatible with the needs of industrial processes. Based on a previously published study [20], UAE was carried out using both a 40 kHz and a 25 kHz acoustic frequency. The extraction trend was monitored over time through the spectrophotometric (λ = 650 nm) measurement of the concentration of released chlorophyll. This allowed us to describe the kinetic of the extraction for the two UAE methods and for the maceration approach (Figure 1). In all conditions, a linear increase in the amount of chlorophyll was observed over time; however, the UAE at 40 kHz seems to be the more efficient, as inferred by the higher level of extracted chlorophyll, and reached a plateau after 40 min. Accordingly, the subsequent analyses were performed on the extracts obtained by subjecting the *C. envidia* leaves to a 40 kHz UAE for 50 min.

### 3.2. NMR-Based Metabolomic Profiling of Escarole

**Figure 2:**
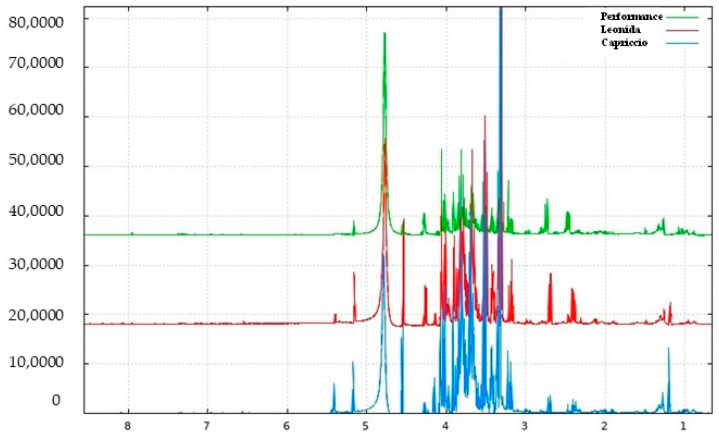
^1^H NMR spectrum of *C. endivia* cultivars from δ_H_ 0.7 to 8.5 ppm.

The escarole extracts obtained from three *C. envidia* cultivars (Capriccio, Performance, and Leonida) underwent metabolic profiling using both NMR and MS-based approaches. Figure 2 shows a representative ^1^H-NMR spectrum of *C. endivia* cultivars extracts. The spectral resonances were assigned based on the corresponding literature data and the 600 MHz version 9 (CL) Chenomx libraries. ^1^H-NMR spectra revealed the presence of compounds mostly belonging to the classes of amino acids, carbohydrates, and phenolic derivatives. Well-resolved and intense signals for low-molecular-weight components were observed in the spectrum zone between 0.5 and 3.0 ppm, and inserted signals were observed between 3.0 and 5.5 ppm and were attributable to sugars. Low-intensity signals between 5.5 to 8.5 ppm were found in the aromatic region.

**Figure 3:**
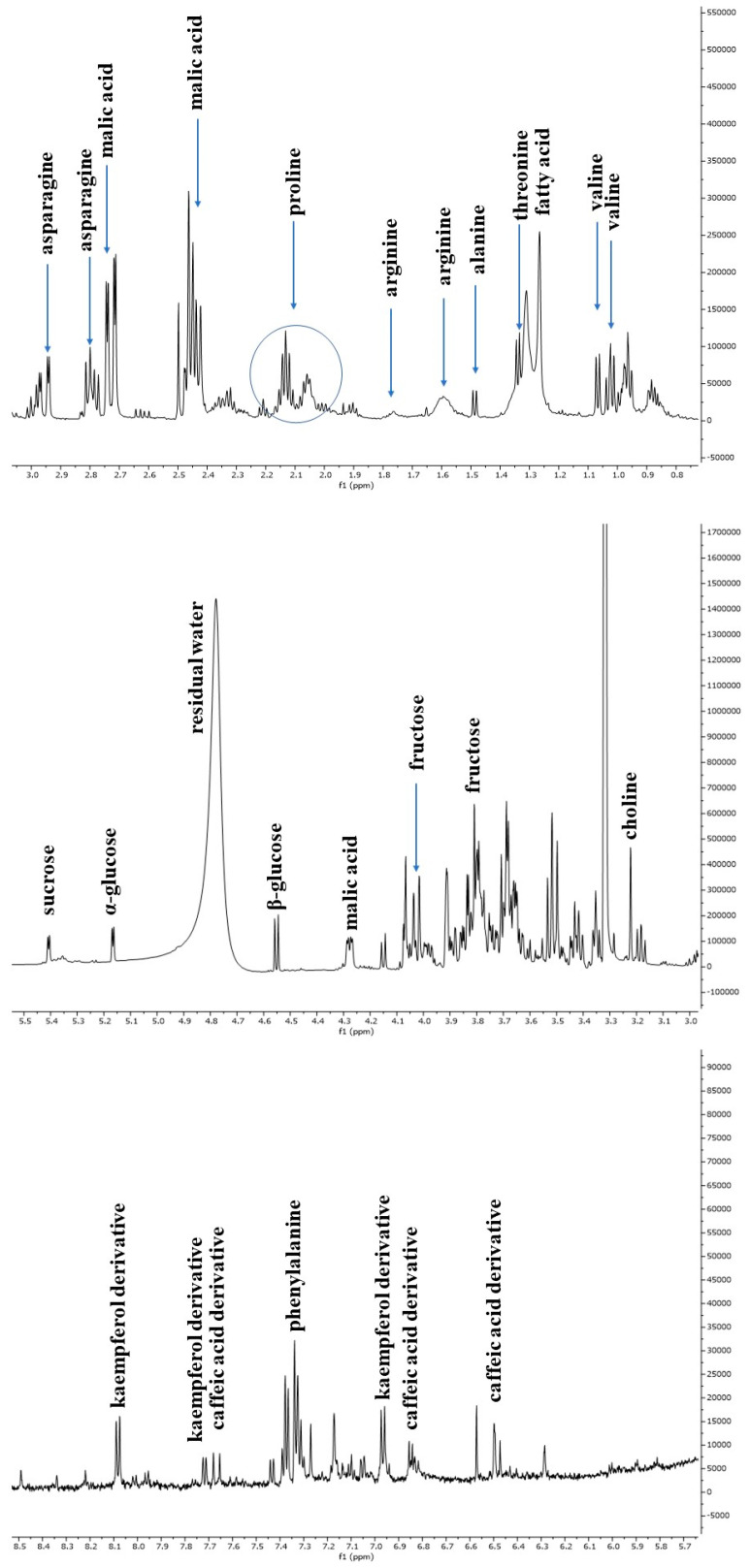
Representative ^1^H NMR spectrum of *C. endivia* ‘Performance’ extract with annotation of the metabolites of the Chenomx 600 MHz custom library (CCL). Regions δ H 0.9–3.1, δ H 3.0–5.5, and δ H 5.6–8.5 were expanded.

The ^1^H-NMR spectrum resonances were assigned according to the corresponding literature data [21], the 600 MHz version 9 (CL), Chenomx libraries, and databases (HMDB [22], BMRB [23]). Considering the signal overlap observed in the spectrum, the identity of several metabolites was further confirmed through 2D NMR (^1^H-^13^C HSQC and COSY) experiments. The chemical shift data for annotated metabolites, with their level of identification according to Sumner et al. [24], are listed in Table 1. The aliphatic region of the spectrum showed resonance corresponding to amino acids, such as valine, threonine and arginine, and malic acid. Among sugars, α-glucose, β-glucose, and sucrose were identified considering the diagnostic resonance of their anomeric protons at 5.16 ppm (d, *J* = 4.4 Hz), 4.54 ppm (d, *J* = 7.9 Hz), and 5.40 ppm (d, *J* = 3.8 Hz), respectively. The downfield region from 6.0 to 8.5 ppm exhibited signals due to the aromatic resonances, mainly due to phenolic acids (chlorogenic and caffeic acid derivatives) and kaempferol derivatives (Figure 3).

### 3.3. LC/MSMS Analysis

#### 3.3.1. Qualitative Analyses

NMR spectroscopy is a promising tool for the plant metabolomic study, but some drawbacks of NMR analysis could be the lack of sensitivity to identify the minor components. Therefore, to obtain an untargeted analysis LC-MS/MS analysis of *C. endivia*, ‘Performance’ leaf extracts were carried out both in positive and negative ion mode, leading to the identification of 35 compounds that belonged to several chemical classes (Table 2). Particularly, six phenolic acids were detected: compound **1** was annotated as caffeoylquinic acid hexoside as it showed a molecular ion at *m/z* 515.1406 [M-H]^−^ and fragment ions at 353 [M-H-162]^−^ and 191 [M-H-162-162]^−^, corresponding to the deprotonated quinic acid. Similarly, the peaks at *m/z* 367.1023 [M-H]^−^ and at *m/z* 337.0935 [M-H]^−^, both displaying their MS^2^ spectra, and the deprotonated quinic acid at *m/z* 191 was assigned to feruloylquinic acid (**13**) and coumaroylquinic acid (**15**), respectively. Chicoric acid (**18**) was identified based on the daughter ion at *m/z* 293 obtained by the loss of caffeic acid residue. Seven compounds were identified as sesquiterpene lactones, characteristic of *Cichorium* ssp., as a result of the determination of their accurate mass and the characteristic fragmentation pattern [25]. Compounds **12** and **2** showed the same molecular ion at *m/z* 277.1070 [M+H]^+^ and fragment ions at *m/z* 241 [M+H^+^-2H_2_O] and 213 [M+H^+^-H_2_O-HCOOH], and were identified as lactucin and one of its isomers, respectively. The identity of **12** was confirmed as it coeluted with the commercial standard. Peaks 5 and 9 (*m/z* 279.1227) were identified as dihydrolactucin and its isomer through to their MS^2^ spectra that showed, as was the case for lactucin, the loss of two water molecules (−36 Da) and formic acid (−64 Da). Dihydro-deoxylactucin (**20**) and deoxylactucin (**27**) were annotated according to their accurate mass value and the presence, in their MS/MS spectra, of fragments similar to those observed for compounds **2**, **5**, **9**, and **12** (Table 2). Compound **29** showed an *m/z* value of 411.1448 [M+H]^+^ and fragment ions at *m/z* 259, due to the loss of 4-hydroxyphenylacetic acid, and *m/z* 213 and was identified as lactucopicrin (Sessa et al., 2000). A similar fragmentation pattern was observed for compound **28**, that showed a molecular ion at *m/z* 413.1598 [M+H]^+^ and was annotated as dihydrolactupicrin. Compounds **17** (*m/z* 223.1328 [M+H]^−^) and **24** (*m/z* 225.1488 [M+H]^−^) were identified as dihydrovomifoliol and vomifoliol, respectively. Moreover, nine compounds (**6**, **7**, **11**, **14**, **16**, **22**, **23**, **25**, **26**) were identified as flavonols. Particularly, compound **6** showed a molecular ion at *m/z* 611.1609 [M+H]^+^ and the loss of two hexose residues in its MS^2^ spectra (−324 Da) and was identified as kaempferol *O*-dihexoside. Similarly, compound **22** (*m/z* 449.1078 [M+H]^+^) showing daughter ion at *m/z* 287 corresponding to the protonated aglycon was annotated as kaempferol *O*-hexoside. Compound **14** (*m/z* 639.1555 [M+H]^−^) was identified as isorhamnetin *O*-dihexoside due to the sequential loss of two hexoside residues in the fragmentation process and a fragment ion at *m/z* 315 corresponding to the protonated aglycon. The loss of a uronic acid moiety (−196 Da) and the presence of the daughter ion at *m/z* 303 [M+H]^+^ in the MS^2^ spectra of compound **16** allowed for its identification as quercetin *O*-uronide. Peak **25** showed a parent ion at *m/z* 535.1069 [M+H]^+^, and the loss of a malonyl-hexoside residue (−248 Da) was identified as kaempferol *O*-malonyl hexoside. The identify of kaempferol *O*-uronide (**23**) was confirmed by the presence of a daughter ion at *m/z* 287, due to the loss of uronic acid in the MS^2^ spectrum and the fragment at 165 in its MS^3^ spectrum. Finally, some polyunsaturated fatty acids were observed at high retention times. In particular, compounds **32** and **33** displayed the same molecular ion at *m/z* 293.2124 [M-H]^−^ and fragments at *m/z* 275 [M-18]^−^ and 231 [M-18-44]^−^ and were identified as two hydroxy octadecatrienoic acid isomers.

#### 3.3.2. Quantitative Analyses

**Figure 4:**
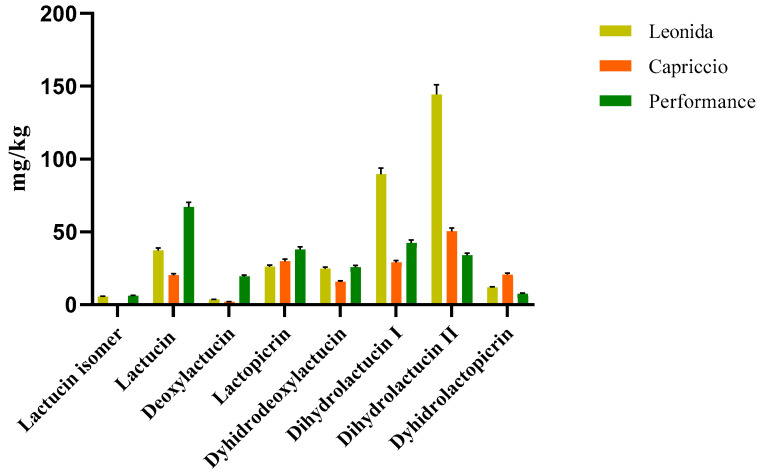
LC-MS/MS-based quantitative analysis of the sesquiterpene lactones retrieved in the leaf extracts of the three cultivars of *C. envidia*. The sesquiterpenes were quantified as lactucin.

Two LC-MS/MS based methods were set to quantify the most representative sesquiterpenes and flavonoids in the leaf extract of the three *C. endivia* cultivars. The sesquiterpenes were quantified as lactucin. The results obtained (Figure 4) highlighted a higher amount of sesquiterpenes in Leonida extract compared to the other two cultivars, while ’Capriccio’ resulted the poorer in these metabolites. In particular, compounds **5** and **9**, (Figure 4) annotated as dihydrolactucin and its isomer, were the most abundant sesquiterpene lactones in the ‘Leonida’.

**Figure 5:**
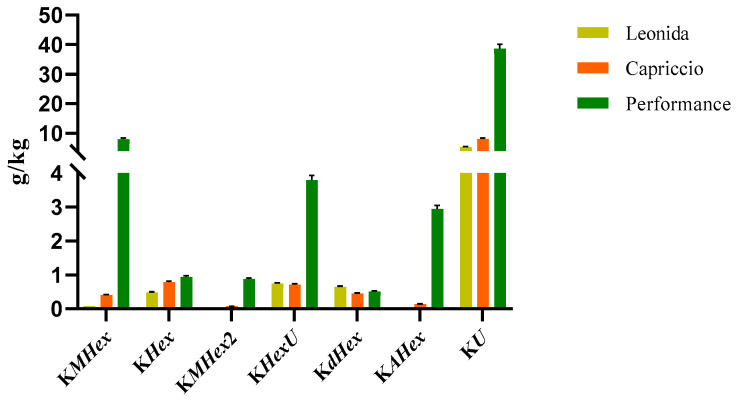
LC-MS/MS-based quantitative analysis of the kaempferol derivatives (KMHex: kaempferol-*O*-malonylhexoside, KHex: kaempferol-*O*-hexoside, KMHex2: kaempferol-*O*-malonylhexoside isomer, KHexU: kaempferol-*O*-hexoside-uronide, KdHex: kaempferol-*O*-dihexoside, KAHex: kaempferol-*O*-acetylhexoside, KU: kaempferol-*O*-uronide) retrieved in the leaf extracts of the three cultivars of *C. envidia*. The compounds were quantified as kaempferol.

Analogously, flavonoids underwent quantitative analysis. In particular, we focused on kaempferol derivatives, which were the most abundant polyphenols in all the three extracts, and we quantified them as kaempferol. The results obtained for these compounds are reported in Figure 5.

According to these data, the ‘Performance’ showed the higher amount of kaempferol derivatives. In particular, the most abundant compounds were those consisting of kaempferol substituted with a malonylhexoside, a hexoside, or an uronide moiety.

### 3.4. Antioxidant Activity

The presence of polyphenols prompted us to investigate the antioxidant activity of ‘Capriccio’, ‘Leonida’, and ‘Performance’ extracts. The radical-scavenger efficacy of the three samples was calculated as Trolox equivalent antioxidant capacity (TEAC) and was stated as the average of the results of at least three independent experiments (Table 3) [17,18,19,26,27,28]. Trolox is an antioxidant used as a standard for reaction with chromogenic radicals, 2,2-diphenyl-1-picrylhydrazyl (DPPH^⋅^) and 2,2′-azino-bis-3-ethylbenzotiazolin-6-sulfonic acid (ABTS^−+^). Although the three extracts showed a significant antioxidant activity regardless of the used assay, some differences were observed among three cultivars. In particular, the extract of ‘Performance’ showed an antioxidant activity that was almost three times higher than those observed for the other two.

**Figure 6:**
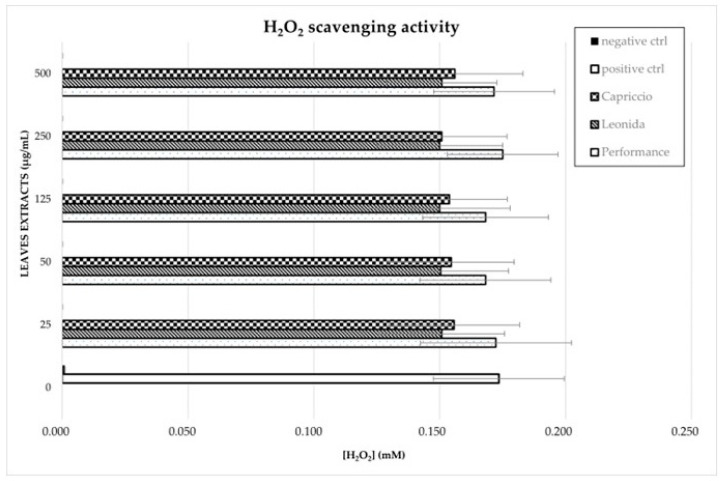
*C. endivia* leaves extracts’ H_2_O_2_ scavenging activity. H_2_O_2_ concentration measured through HRP enzymatic assay after incubation with different concentrations of *C. endivia* extracts is shown. Positive control is represented by the enzymatic assay in the absence of natural extracts and negative control was performed with a previous incubation of the reaction mixture with catalase to obtain H_2_O_2_ degradation.

Finally, the H_2_O_2_ scavenging activity of natural extracts was also evaluated using the horseradish peroxidase (HRP) assay as described in Materials and Methods. None of the *C. endivia* extracts displayed a significant peroxide scavenging activity in the in vitro conditions tested. A slight decrease in H_2_O_2_ concentration was observed after incubation in the presence of Leonida and Capriccio extracts, as compared to Performance, but it was not statistically significant (Figure 6).

### 3.5. In Cell Antioxidant Activity

**Figure 7:**
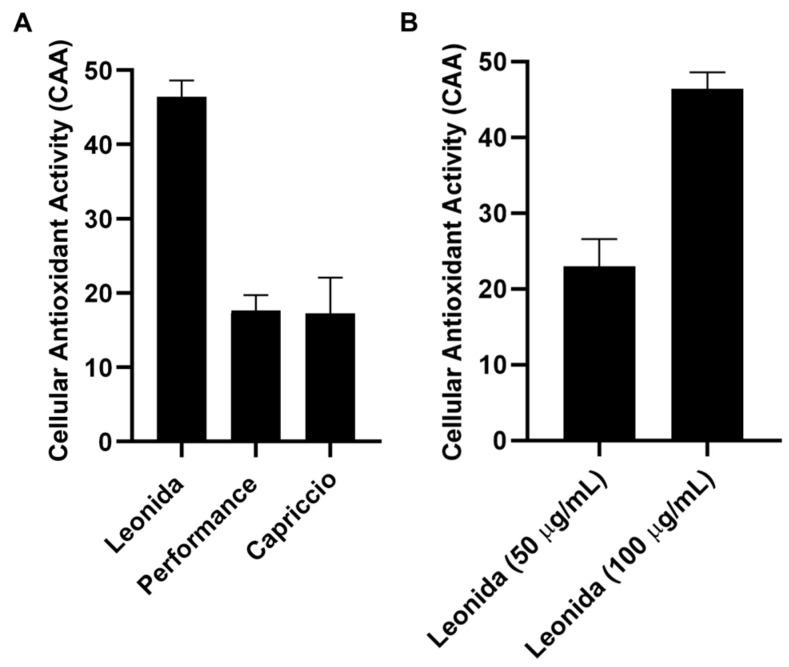
(**A**) Cellular antioxidant activity (CAA) of ‘Leonida’, ‘Performance’, and ‘Capriccio’ on THP-1-derived macrophage M0 cells at the concentration of 100 μg/mL. (**B**) Cellular Antioxidant Activity (CAA) of C. endivia cultivars ‘Leonida’ on THP-1-derived macrophage M0 cells at the concentration of 50–100 μg/mL.

The cellular antioxidant activity (CAA) of the three extracts was assessed in M0 macrophages cells. These cells were subjected to a 1 h treatment with each extract (100 µg/mL) or were left untreated, incubated with a radical initiator, and the production of radical species was monitored for 60 min. Comparing the effects of the different extracts (Figure 6) showed that those from ‘Performance’ and ‘Capriccio’ did not produce any significant antioxidant activity, while cells treated with Leonida extract reduced radical production of about 50% in regards to the untreated cells. ‘Leonida’ extract was also tested at a concentration of 50 µg/mL, confirming its antioxidant effect, and showing some dose-dependent behaviour (Figure 7).

**Figure 8:**
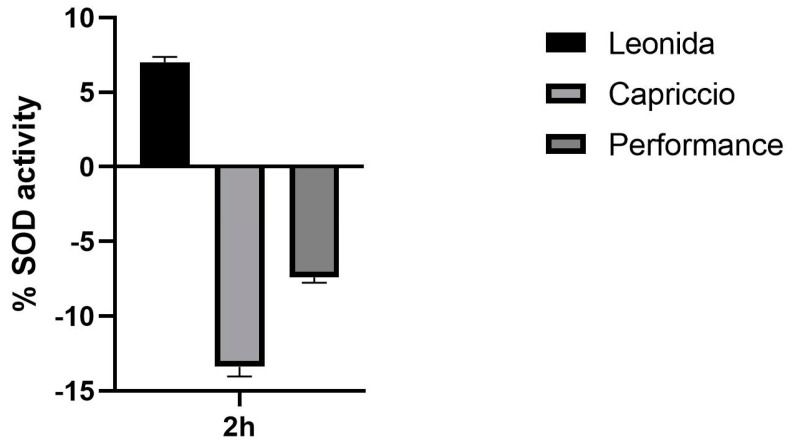
Effect of ‘Leonida’, ‘Performance’, and ‘Capriccio’ extracts on SOD enzymatic activity in THP-1-derived macrophage M0 cells at a concentration of 100 μg/mL.

In an attempt to elucidate the mechanism underlying this antioxidant action of Leonida, the effect of the three extracts on SOD activity was investigated. At this end, a colorimetric assay was used to measure the activities of all SOD types, including Cu/Zn-, Mn-, and Fe-dependent enzymes. Interestingly, a 2 h treatment of the cells with 100 µg/mL of Leonida extract induced a significant increase in the SOD catalytic activity, while Capriccio and Performance extracts were ineffective (Figure 8).

**Figure 9:**
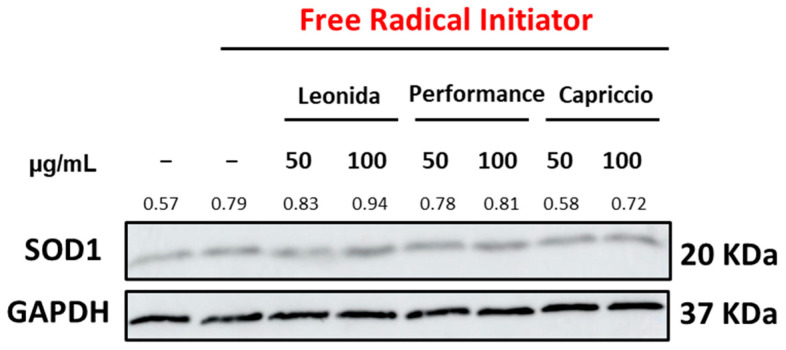
Western blot of ‘Leonida’, ‘Performance’, and ‘Capriccio’ tested at the concentration of 50–100 µg/mL on THP-1-derived M0 in the presence of Free Radical Initiator.

Furthermore, a western blot analysis indicated that a 1h pre-treatment of the cells with ‘Leonida’ extract induced a slight increase of SOD1 expression level under oxidative stress conditions (Figure 9).

Conversely, none of the three extracts were able to significantly modulate catalase activity in vitro. Catalase activity was evaluated by assessing the H_2_O_2_ degradation catalysed by the enzyme, as described in Materials and Methods. Data shown in Table 4 highlighted that catalase activity in vitro was not hampered by the incubation in the presence of 500 μg/mL of *C. endivia* leaf extracts. In fact, residual peroxide concentration, quantified by a coupled HRP assay, did not increase in the presence of natural extracts, thus indicating no inhibition in the peroxide conversion by catalase.

Given the chemical complexity of the tested samples, the antioxidant effect exerted by cells treatment with ‘Leonida’ extract may depend on multiple mechanisms. However, our result suggested that it is at least partially mediated by an enhancement of SOD activity.

### 3.6. Anti-Inflammatory Activity

**Figure 10:**
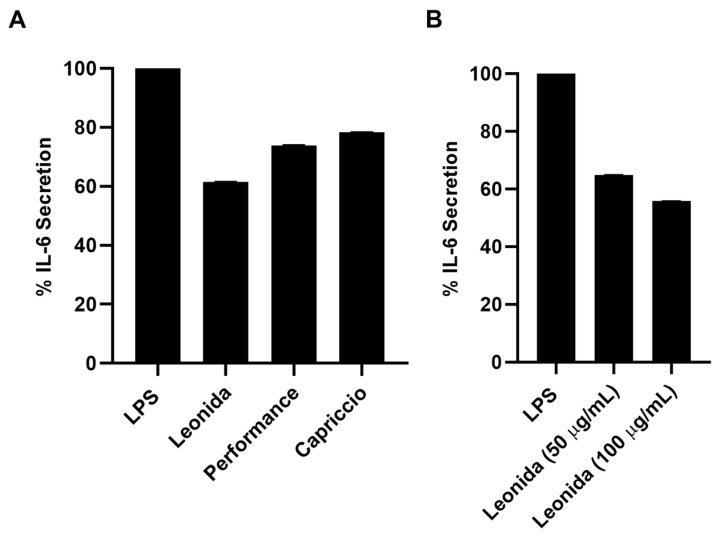
(**A**) Percentage of secretion of IL-6 from LPS-stimulated THP-1-derived macrophage M0 cells after 24 h of treatment with ‘Leonida’, ‘Performance’, and ‘Capriccio’ at the concentration of 100 μg/mL. (**B**) Percentage of secretion of IL-6 from LPS-stimulated THP-1-derived macrophage M0 cells after 24 h of treatment with ‘Leonida’ at the concentration of 50 and 100 μg/mL.

Given the presence of non-negligible concentrations of sesquiterpenes, the anti-inflammatory effect of the extracts of the three cultivars of *C. envidia* was also tested. Plant sesquiterpenes account for several biological effects [14,29,30]. In particular, the cytotoxicity and the ability to modulate different pathways involved in the inflammatory response has been described for these molecules [31,32]. Therefore, all the extracts were assayed by the MTT test on THP-1-derived M0 macrophages to verify they did not affect cell viability. Since all the extracts were not cytotoxic, their anti-inflammatory property was investigated on LPS-stimulated M0 macrophages that underwent a 24-h treatment with 100 μg/mL of each extract. *C. envidia* ‘Performance’ and ‘Capriccio’ showed moderate anti-inflammatory activity, whereas the ‘Leonida’ extract was able to induce a reduction of IL-6 secretion of about 40% (Figure 10A). Furthermore, for the latter, an inhibition of IL-6 secretion of about 60% was observed at 50 μg/mL (Figure 10B).

## 4. Discussion

Plants of *Cichorium* genus are well known due to their beneficial properties. Many studies were conducted on specialized metabolites such as coumarins, flavonoids, sesquiterpenoids, triterpenoids, steroids, organic acids, and other chemical constituents; additionally, biological effects such as antioxidant and anti-inflammation were reported. *Cichorium endivia* L. belongs to this genus and is one of the most consumed salads in the world.

In Italy, the area cultivated with curly or smooth-leafed escarole is over 6800 hectares with a total production of over 152,800 tons, most of which (about 100,000 tons) come from the southern regions. Campania, with a production of 27,895 tons [33], is the first producer in Italy of this protected crop, and this cultivation represents an important economic activity for this region. Particularly, in Salerno areas, escarole production is 252,000 quintals out of 312,000 quintals of the Campania region [33].

On this basis, our study aimed to assess the suitability of *C. endivia*-derived waste for its valorisation in order to include these products in the circular economy processes of Salerno territory. Waste valorisation, the process of turning waste into beneficial products [34], is a challenging process to address the administration of waste materials. For this purpose, we have implemented multiplatform approaches to achieve a description of the *C. endivia* leaf metabolome. Specifically, we used nuclear magnetic resonance (NMR) and mass spectrometry (MS) in combination with biological and biochemical assays. The application of NMR technique in the quality control of food products has lately employed in the “foodomics” science. With the aim of defining the most relevant components of nutritional interest from *C. endivia* var *latifolium* ‘Performance’, *C. endivia* var *latifolium* ‘Leonida’, and *C. endivia* var *crispum* ‘Capriccio’, a high-resolution NMR-based protocol was set up; the obtained data were analysed using the Chenomx NMR Suite v9.0 software. The Chenomx Spectral Reference Libraries were developed from thousands of references registered in a pH range 4–9 and NMR field strengths from 400 MHz through 800 MHz. The characterization of the secondary metabolites of *C. endivia* cultivars was carried out through the study of the phytochemical profile obtained by LC-HRMS/MS analysis, which highlighted the presence of flavonoids, phenolic acids, and lactone sesquiterpenes typical of the species. Based on the composition of the analysed extracts, we assayed their radical scavenger efficacy and in-cell antioxidant and anti-inflammatory potential. As expected, the presence of polyphenols in the extracts provided effective anti-radical activity, which was higher for the sample that was richer in kaempferol derivatives (‘Performance’). Conversely, only the extract of ‘Leonida’ produced a significant antioxidant effect at a cellular level. This unexpected result may depend on the low bioavailability of the glycosylated polyphenols [35], which were very abundant in the ‘Performance’ extract. On the other hand, the presence of the ‘Leonida’ extract in compounds was able to stimulate anti-oxidative cellular pathways. Sesquiterpenoids, which were abundant in this extract, have been recently shown to activate several intracellular mechanisms, resulting in a protection from oxidative insults [36]. An indirect confirmation of this hypothesis was provided by an analysis of the anti-inflammatory activity of the extracts. Additionally, in this case, the extract that showed the most significant effect was ‘Leonida’, thus corroborating the idea that the higher amount of sesquiterpene lactones retrieved in these extracts could correlate with a more significant ability to interfere with cellular biochemistry. The obtained results can be useful to advance the knowledge on the phytochemical composition and biological properties of these scarola cultivars. *C. endivia* is a versatile vegetable, containing significant amounts of proteins, carbohydrates, minerals, and phytoconstituents, and it can contribute to the healthy nutrition of animals and people. This investigation established that discarded leaves possess interesting antioxidant and anti-inflammatory properties and are thus convertible in healthy product preparations.

## Figures and Tables

**Table 1:** Compounds and ^1^H chemical shifts identified by 600 MHz ^1^H-NMR for escaroles (600 MHz).

Compounds	Chemical Shift (ppm) Multiplicity (*J* in Hz)	MSI Status^a^	Identification Confirmation
Valine	1.07 d (*J* = 7.2); 1.02 d (*J* = 7.2)	2	^1^H-^13^C HSQC
Threonine	1.34 d (*J* = 6.5)	2	^1^H-^13^C HSQC
Alanine	1.49 d (*J* = 7.30)	2	^1^H-^13^C HSQC
Malic Acid	2.46 dd, (*J* = 15.6, 8.3); 2.74 dd (*J* = 16.0, 3.5)	2	^1^H-^13^C HSQC
Arginine	1.59 m; 1.77 m	2	^1^H-^13^C HSQC
Asparagine	2.95 dd (*J* = 14.7, 3.9); 2.78 dd (*J* = 16.8, 9.0)	2	^1^H-^13^C HSQC
Choline	3.2 s	2	^1^H-^13^C HSQC
α-Glucose	5.16 d (*J* = 4.40)	1	^1^H-^13^C HSQC, Spike
β-Glucose	4.54 d (*J* = 7.9)	1	^1^H-^13^C HSQC Spike
Sucrose	5.40 d (*J* = 3.8)	1	^1^H-^13^C HSQC, Spike
Fructose	4.02 dd (*J* = 12.3, 1.3)	2	^1^H-^13^C HSQC
α-Galactose	5.2 d (*J* = 3.4)	2	^1^H-^13^C HSQC
Phenylalanine	7.37 m	2	^1^H-^13^C HSQC
Fatty acid	1.27 s	3	^1^H-^13^C HSQC
Caffeic acid derivative	7.70 d (*J* = 16.0); 6.48 d (*J* = 16.0)	3	^1^H-^13^C HSQC
Kaempferol derivative	8.08 d (*J* = 8.7); 6.97 d (*J* = 8.7)	3	^1^H-^13^C HSQC

^a^ MSI level of identification based on Sumner et al., 2007 [24].

**Table 2:** Secondary metabolites identified in leaf extract *C. endivia* ‘Performance’ by LC-MS/MS analysis.

Pek	R_t_ (Min)	[M+H]^+^	[M-H]^−^	Fragment Ions	Compound Identity	MSI Status ^a^
1	11.66		515.1406	353 [M-162]^−^ 191 [M-162-162]^−^	Caffeoylquinic acid-*O*-hexoside	2
2	12.68	277.1070		241 [M-36]^+^ 213 [M-64]^+^ 185 [M-92]^+^	Lactucin isomer	2
3	13.62	341.0867		179 [M-162]^+^	Aesculin	1
4	15.69	355.1023		163 [M-192]^+^	Chlorogenic acid	2
5	16.04	279.1227		243 [M-36]^+^ 215 [M-64]^+^ 187 [M-92]^+^	Dihydrolactucin	2
6	17.17	611.1609		287 [M-324]^+^	Kaempferol-*O*-dihexoside	2
7	17.26	625.1401		287 [M-338]^+^	Kaempferol-*O*-hexoside-uronide	2
8	17.56	389.2177		371 [M-18]^+^	Dihydroroseoside	2
9	17.65	279.1227		243 [M-36]^+^ 215 [M-64]^+^ 187 [M-92]^+^	Dihydrolactucin isomer	2
10	17.77	387.2015		369 [M-18]^+^	Roseoside	1
11	18.35	697.1608		287 [M-410]^+^	Kaempferol-*O*-malonyldihexoside	2
12	18.68	277.1070		241 [M-36]^+^ 213 [M-64]^+^ 185 [M-92]^+^	Lactucin	1
13	19.19		367.1023	191 [M-176]^−^ 173 [M-176-18]^−^	Feruloylquinic acid	2
14	19.46		639.1555	477 [M-162]^−^ 315 [M-162-162]^−^	Isorhamnetina-*O*-dihexoside	2
15	19.69		337.0935	191 [M-196]^−^	Coumaroylquinic acid	2
16	21.86	479.0820		303 [M-176]^+^	Quercetin-*O*-uronide	2
17	22.12	223.1328		205 [M-18]^+^ 187 [M-36]^+^ 105 [M-118]^+^	Dihydro-vomifoliol	2
18	22.64		473.0733	293 [M-180]^−^	Chicoric acid	2
19	22.68	197.1172		179 [M-26]^+^ 161 [M-36]^+^ 135 [M-62]^+^	Loliolide	1
20	23.02	263.1277		245 [M-18]^+^ 217 [M-46]^+^ 189 [M-74]^+^	Dihydro-deoxylactucin	2
21	23.14	517.1346		163 [M-354]^+^	Dicaffeoylquinic acid	2
22	23.21	449.1078		287 [M-162]^+^	Kaempferol-*O*-hexoside	2
23	23.33	463.0869		287 [M-176]^+^	Kaempferol-*O*-uronide	2
24	23.95	225.1488		207 [M-18]^+^	Vomifoliol	2
25	24.76	535.1069		287 [M-248]^+^	Kaempferol-*O* malonylhexoside	2
26	25.08		489.1049	285 [M-204]^−^	Kaempferol-*O*-acetylhexoside	2
27	30.12	261.1121		243 [M-18]^+^ 215 [M-46]^+^ 187 [M-74]^+^	Deoxyilactucin	2
28	30.13	413.1598		261 [M-152]^−^ 215 [M-198]^−^	Dihydrolactucopicrin	2
29	30.19	411.1442		259 [M-152]^−^ 213 [M-198]^−^ 185 [M-226]^−^	Lactucopicrin	2
30	31.14		327.2181	309 [M-18]^−^ 291 [M-18]^−^ 229 [M-98]^−^ 211 [M-98-18]^−^	Trihydroxy-octadecadienoic acid	2
31	32.87		329.2339	311 [M-18]^−^ 293 [M-18]^−^	Trihydroxy-octadecadienoic acid	2
32	46		293.2124	275 [M-18]^−^ 231 [M-18-44]^−^	Hydroxy-octadecatrienoic acid	2
33	46.21		293.2124	275 [M-18]^−^ 231 [M-18-44]^−^	Hydroxy-octadecatrienoic acid isomer	2
34	48.67		277.2173	259 [M-18]^−^	Octadecatrienoic acid	2
35	49.85		279.2328	261 [M-18]^−^	Octadecadienoic acid	2

^a^ MSI level of identification based on Sumner et al., 2007 [24].

**Table 3:** Antioxidant activities of leaves extract of three cultivars—Leonida, Performance, and Capriccio.

Samples	DPPH TEAC mM	ABTS TEAC mM
*C. endivia* var *latifolium*. ‘Performance’	2.51 ± 0.25	3.49 ± 0.28
*C. endivia* var *latifolium* ‘Leonida’	0.60 ± 0.04	0.78 ± 0.07
*C. endivia* var *crispum*. ‘Capriccio’	0.95 ± 0.05	1.14 ± 0.22

Values are expressed as means ± standard deviation (*n* = 3). TEAC (Trolox equivalent antioxidant capacity) is expressed as mM Trolox equivalent. DPPH: 2,2-diphenyl-1-picrylhydrazyl. ABTS: 2,20-azino-bis (3-ethylbenzothiazoline-6-sulphonic acid).

**Table 4:** *C. endivia* leaf extracts and their catalase activity inhibition. The H_2_O_2_ concentration in the reaction mixture was calculated with a coupled enzymatic assay using HRP peroxidase and pyrogallol conversion in purpurgallin. Purpurgallin absorbance at 420 nm at the end of the reactions is indicated in Table 4.

Samples	Abs 420 nm	H_2_O_2_ Concentration (mM)
Negative control (without catalase)	2.200	0.625 ± 0.1
Positive control (30 U catalase)	0.002	0.001
*C. endivia* var *latifolium*. ‘Performance’	0.003	0.001
*C. endivia* var *latifolium* ‘Leonida‘	0.002	0.001
*C. endivia* var *crispum*. ‘Capriccio’	0.002	0.001

## Data Availability

Data are contained within the article.

## References

[1] Sareedenchai V., Zidorn C. (2015). Flavonoids as chemosystematic markers in the tribe Cichorieae of the Asteraceae. Biochem. Syst. Ecol..

[2] Hegazy A.K., Ezzat S.M., Qasem I.B., Ali-Shtayeh M.S., Basalah M.O., Ali H.M., Hatamleh A.A. (2015). Diversity of active constituents in *Cichorium endivia* and *Cynara cornigera* extracts. Acta Biol. Hung..

[3] Llorach R., Martinez-Sanchez A., Tomas-Barberan F.A., Gil M.I., Ferreres F. (2008). Characterization of polyphenols and antioxidant properties of five lettuce varieties and escarole. Food Chem..

[4] Warashina T., Miyase T. (2008). Sesquiterpenes from the Roots of *Cichorium endivia*. Chem. Pharm. Bull..

[5] Molan A.L., Duncan A.J., Barryand T.N., McNabb W.C. (2003). Effect of condensed tannins and sesquiterpene lactones extracted from chicory on the motility of larvae of deer lungworm and gastrointestinal nematodes. Parasitol. Int..

[6] Muthusamy V.S., Anand S., Sangeetha K.N., Sujatha S., Arun B., Lakshami B.S. (2008). Tannins present in Cichorium intybus enhance glucose uptake and inhibit adipogenesis in 3T3-L1 adipocytes through PTP1B inhibition. Chem. Biol. Interact..

[7] Innocenti M., Gallori S., Giaccherini C., Ieri F., Vincieri F.F., Mulinacci N. (2005). Evaluation of phenolic content in the aerial parts different varieties of *Cichorium intybus* L.. J. Agric. Food Chem..

[8] Perović J., Šaponjac V.T., Kojić J., Krulj J., Moreno D.A., García-Viguera C., Bodroža-Solarov M., Ilić N. (2021). Chicory (*Cichorium intybus* L.) as a food ingredient–Nutritional composition, bioactivity, safety, and health claims: A review. Food Chem..

[9] Abu-Reidah I.M., Contreras M.M., Arráez-Román D., Segura-Carretero A., Fernández-Gutiérrez A. (2013). Reversed-phase ultra-high- liquid chromatography coupled to electrospray ionization-quadrupole-time-of-flight mass spectrometry as a powerful tool for metabolic profiling of vegetables: *Lactuca sativa* as an example of its application. J. Chromatogr. A.

[10] Atta A.H., Elkoly T.A., Mouneir S.M., Kamel G., Alwabel N.A., Zaher S. (2010). Hepatoprotective effect of methanolic extracts of *Zingiber officinale* and *Cichorium intybus*. Indian J. Pharm. Sci..

[11] Lucchin M., Varotto S., Barcaccia G., Parrini P., Prohens J., Nuez F. (2008). Chicory and Endive. Vegetables I: Asteraceae, Brassicaceae Chenopodi-Caceae.

[12] Testone G., Mele G., Di Giacomo E., Tenore G.C., Gonnella M., Nicolodi C., Frugis G., Iannelli M.A., Arnesi G., Schiappa A. (2019). Transcriptome driven characterization of curly- and smooth-leafed endives reveals molecular differences in the sesquiterpenoid pathway. Hortic. Res..

[13] Wesolowska A., Nikiforuk A., Michalska K., Kisiel W., Chojnacka-Wojcik E. (2006). Analgesic and sedative activities of lactucin and some lactucin-like guaianolides in mice. J. Ethnopharmacol..

[14] Matos M.S., Anastácio J.D., Nunes dos Santos C. (2021). Sesquiterpene lactones: Promising natural compounds to fight inflammation. Pharmaceutics.

[15] Ahmed N., Thompson S., Turchini G.M. (2020). Organic aquaculture productivity, environmental sustainability, and food security: Insights from organic agriculture. Food Secur..

[16] Vertakova Y.V., Plotnikov V.A. (2019). The integrated approach to sustainable development: The case of energy efficiency and solid waste management. Int. J. Energy Econ. Policy.

[17] Nariya P.B., Bhalodia N.R., Shukla V.J., Acharya R., Nariya M.B. (2013). In vitro evaluation of antioxidant activity of Cordia dichotoma (Forst f.) bark. Ayu.

[18] Huang D., Ou B., Prior R.L. (2005). The chemistry behind antioxidant capacity assay. J. Agric. Food Chem..

[19] Wolfe K.L., Liu R.H. (2007). Cellular Antioxidant Activity (CAA) assay for assessing antioxidants, foods, and dietary supplements. J. Agric. Food Chem..

[20] Zhang L., Zhou C., Wang B., Yagoub A.E.G.A., Ma H., Zhang X., Wu M. (2017). Study of ultrasonic cavitation during extraction of the peanut oil at varying frequencies. Ultrason. Sonochem..

[21] Sobolev A.P., Brosio E., Gianferri R., Segre A.L. (2005). Metabolic profile of lettuce leaves by high-field NMR spectra. Magn. Reson. Chem..

[22] Wishart D.S., Tzur D., Knox C., Eisner R., Guo A.C., Young N., Cheng D., Jewell K., Arndt D., Sawhney S. (2007). HMDB: The Human Metabolome Database. Nucleic Acids Res..

[23] Markley J.L., Anderson M.E., Cui Q., Eghbalnia H.R., Lewis I.A., Hegeman A.D., Li J., Schulte C.F., Sussman M.R., Westler W.M. (2007). New bioinformatics resources for metabolomics. Pac. Symp. Biocomput..

[24] Sumner L.W., Amberg A., Barrett D., Beale M.H., Beger R., Daykin C.A., Fan T.W.M., Fiehn O., Goodacre R., Griffin J.L. (2007). Proposed minimum reporting standards for chemical analysis. Metabolomics.

[25] Graziani G., Ferracane R., Sambo P., Santagata S., Nicoletto C., Fogliano V. (2015). Profiling chicory sesquiterpene lactones by high resolution mass spectrometry. Food Res. Int..

[26] Abbas Z.K., Saggu S., Sakeran M.I., Zidan N., Rehman H., Ansari A.A. (2015). Phytochemical, antioxidant and mineral composition of hydroalcoholic extract of chicory (*Cichorium intybus* L.) leaves. Saudi J. Biol. Sci..

[27] Zhong Y., Shahidi F., Shadidi F. (2015). Methods for the assessment of antioxidant activity in foods. Handbook of Antioxidants for Food Preservation.

[28] Jiménez I., Sánchez-Moreno C., Saura-Calixto F. (2000). Evaluation of free radical scavenging of dietary carotenoids by the stable radical 2,2-diphenyl-1-picrylhydrazyl. J. Sci. Food Agric..

[29] Sokovic M., Ciric A., Glamoclija J., Skaltsa H. (2017). Biological Activities of Sesquiterpene Lactones Isolated from the *Genus centaurea* L. (Asteraceae). Curr. Pharm. Des..

[30] Kreuger M.R., Grootjans S., Biavatti M.W., Vandenabeele P., D’Herde K. (2012). Sesquiterpene lactones as drugs with multiple targets in cancer treatment: Focus on parthenolide. Anti-Cancer Drugs.

[31] Quintana J., Estévez F. (2018). Recent Advances on Cytotoxic Sesquiterpene Lactones. Curr. Pharm. Des..

[32] Paço A., Brás T., Santos J.O., Sampaio P., Gomes A.C., Duarte M.F. (2022). Anti-inflammatory and immunoregulatory action of sesquiterpene lactones. Molecules.

[33] Crops: Areas and Production—Overall Data—Provinces. http://dati.istat.it/Index.aspx?QueryId=37850#.

[34] Arancon R.A.D., Lin C.S.K., Chan K.M., Kwan T.H., Luque R. (2013). Advances on waste valorization: New horizons for a more sustainable society. Energy Sci. Eng..

[35] Stefaniu A., Pirvu L.C. (2022). In Silico Study Approach on a Series of 50 Polyphenolic Compounds in Plants; A Comparison on the Bioavailability and Bioactivity Data. Molecules.

[36] Li Y., Liu J., Wu Y., Li Y., Guo F. (2022). Guaiane-type sesquiterpenes from Curcumawenyujin. Phytochemistry.

